# NMR data of a Grubbs 2^nd^ generation catalyst *p*-cresolate derivative

**DOI:** 10.1016/j.dib.2020.106634

**Published:** 2020-12-08

**Authors:** M.R. Swart, Barend C.B. Bezuidenhoudt, C. Marais, E. Erasmus

**Affiliations:** Department of Chemistry, University of the Free State, Bloemfontein, 9300, Republic of South Africa

**Keywords:** Grubbs 2^nd^ generation catalyst, *p*-cresol derivatives, 1D and 2D NMR

## Abstract

The data presented in this article is related to the research article entitled “Spectroscopic characterisation of Grubbs 2^nd^ generation catalyst and its *p*-cresol derivatives” (Swart *et al.* 2021). The 1D and 2D NMR characterisation data of the *p-*cresol derivative of the Grubbs 2^nd^ generation catalyst, where one of the chloride ligands is replaced by the *p*-cresolate to form a Ru-O coordination compound (**3**) is reported. The characterization data include information obtained from ^1^H, ^13^C, Heteronuclear Single Quantum Coherence (HSQC), Heteronuclear Multiple Bond Correlation (HMBC), Homonuclear Correlation Spectroscopy (COSY), Nuclear Overhauser Effect (NOE) and Distortionless Enhancement by Polarization Transfer (DEPT) NMR spectroscopy.

## Specifications Table

**Table:** 

Subject	Chemistry
Specific subject area	Homogeneous catalysis, spectroscopic characterisation
Type of data	Table Figure
How data were acquired	Bruker AVANCE II 600 FT NMR spectrometer, 400 MHz AVANCE III spectrometer, Microsoft Excel 2016
Data format	Raw Analysed
Parameters for data collection	The NMR spectra were recorded using following parameters: Solvent: CDCl_3_ Temperature (K): 278.15 Spectrometer frequency (MHz): 600.28 (^1^H) & 150.95 (^13^C) Number of scans: 8 (HSQC); 8 (HMBC); 8 (COSY); 8 (NOE) & 4 (DEPT) Relaxation delay(sec): 1.5 Acquisition time (sec): HSQC: AQ (^1^H) = 0.0655; AQ (^13^C) = 0.00486; HMBC: AQ (^1^H) = 0.131; AQ (^13^C) = 0.00486 COSY: AQ (^1^H) = 00655; AQ (^1^H) = 0.033 NOE: AQ (^1^H) = 0.131; AQ (^1^H) = 0.016 DEPT: AQ (^13^C) = 0.612 Spectral width: HSQC: SW (^1^H) = 26.0 ppm / 15 625 Hz; SW (^13^C) = 348.63 ppm / 52 631 Hz HMBC: SW (^1^H) = 26.0 ppm / 15 625 Hz SW (^13^C) = 348.63 ppm / 52 631 Hz COSY: SW (^1^H) = 26.0 ppm / 15 625 Hz SW (^1^H) = 25.9 ppm / 15607 Hz NOE: SW (^1^H) = 26.0 ppm / 15 625 Hz SW (^1^H) = 25.9 ppm / 15607 Hz DEPT: SW (^13^C) = 354.85 ppm / 53571 Hz
Description of data collection	NMR: Chemical shifts were shown as *δ*-values with reference to tetramethylsilane (TMS) as an internal standard.
Data source location	University of the Free State, Bloemfontein, South Africa Latitude: -29.110028° Longitude: 26185706°
Data accessibility	See Supplementary Information.
Related research article	M.R. Swart, Barend C.B. Bezuidenhoudt, C. Marais, E. Erasmus, Spectroscopic characterisation of Grubbs 2^nd^ generation catalyst and its *p*-cresol derivatives, Inorganica Chimica Acta, 2021, 514, 120001, doi.org/10.1016/j.ica.2020.120001 [1].

## Value of the Data

• The data represents the NMR characterization of the Grubbs 2^nd^ generation catalyst and *p-* cresol derivatives thereof.
• The data can be useful to researchers interested in improving Grubbs 2^nd^ generation catalyst for a variety of metathesis reactions.
• Our data contributes to the characterization of the Grubbs 2^nd^ generation catalyst and its *p*-cresol derivatives.
• The data will be useful for the modification of catalysts towards improved metathesis.

## 1 Data Description

**Fig. 1:**
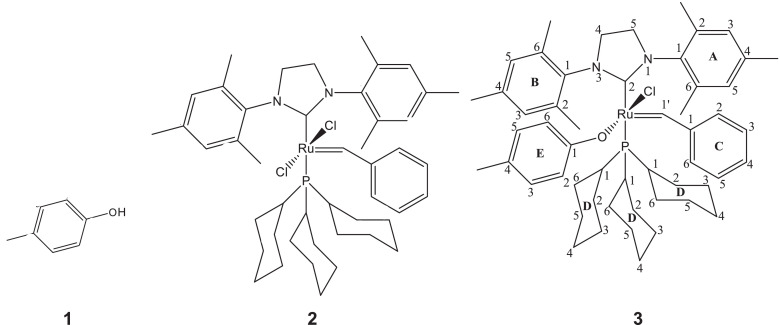
The structures of p-cresol **(1)**, Grubbs 2^nd^ generation catalyst **(2)**, and the modified Grubbs 2^nd^ generation- p-cresolate catalyst, **3**. The structure of **3**, shows the numerical labels of the carbon atoms used to indicate their NMR positions reported in Table 1.

**Fig. 2:**
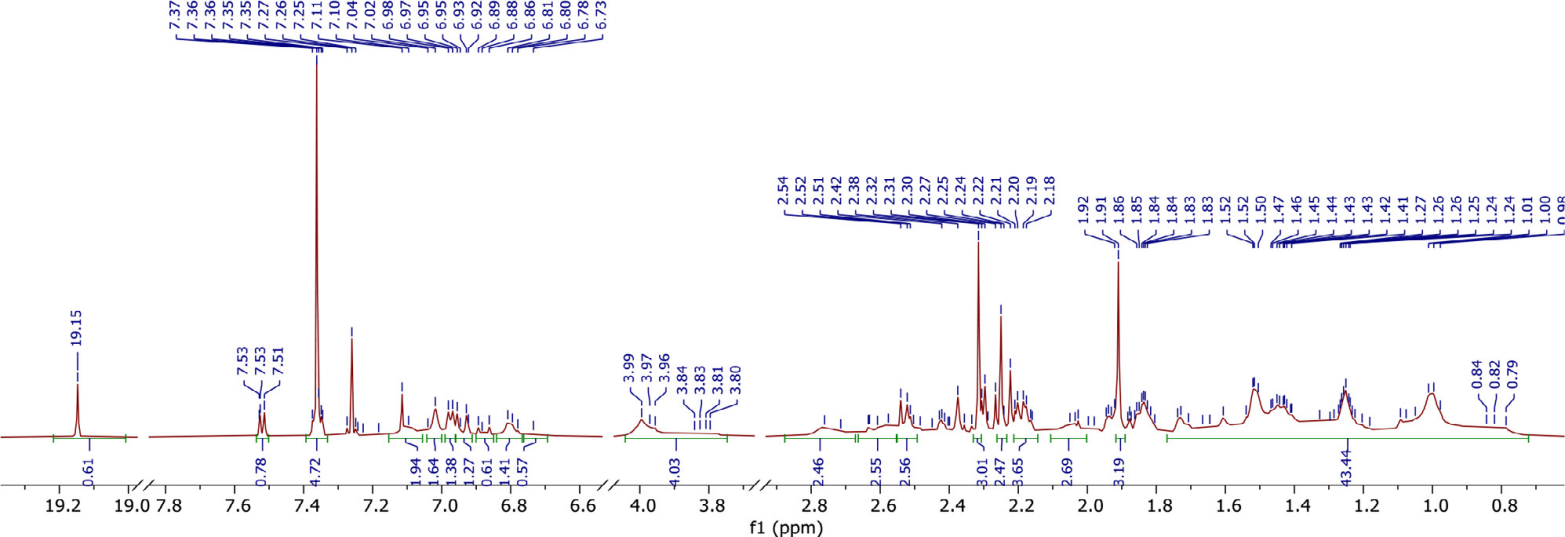
The ^1^H NMR spectra of **3** measured in CDCl_3_.

**Fig. 3:**
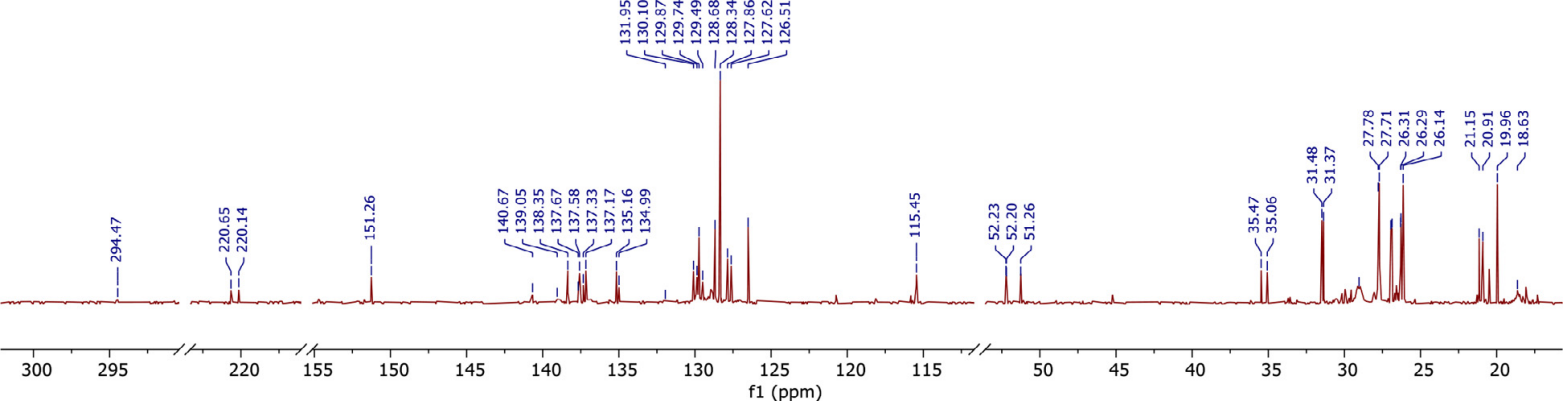
The ^13^C NMR spectra of **3** measured in CDCl_3_.

**Fig. 4:**
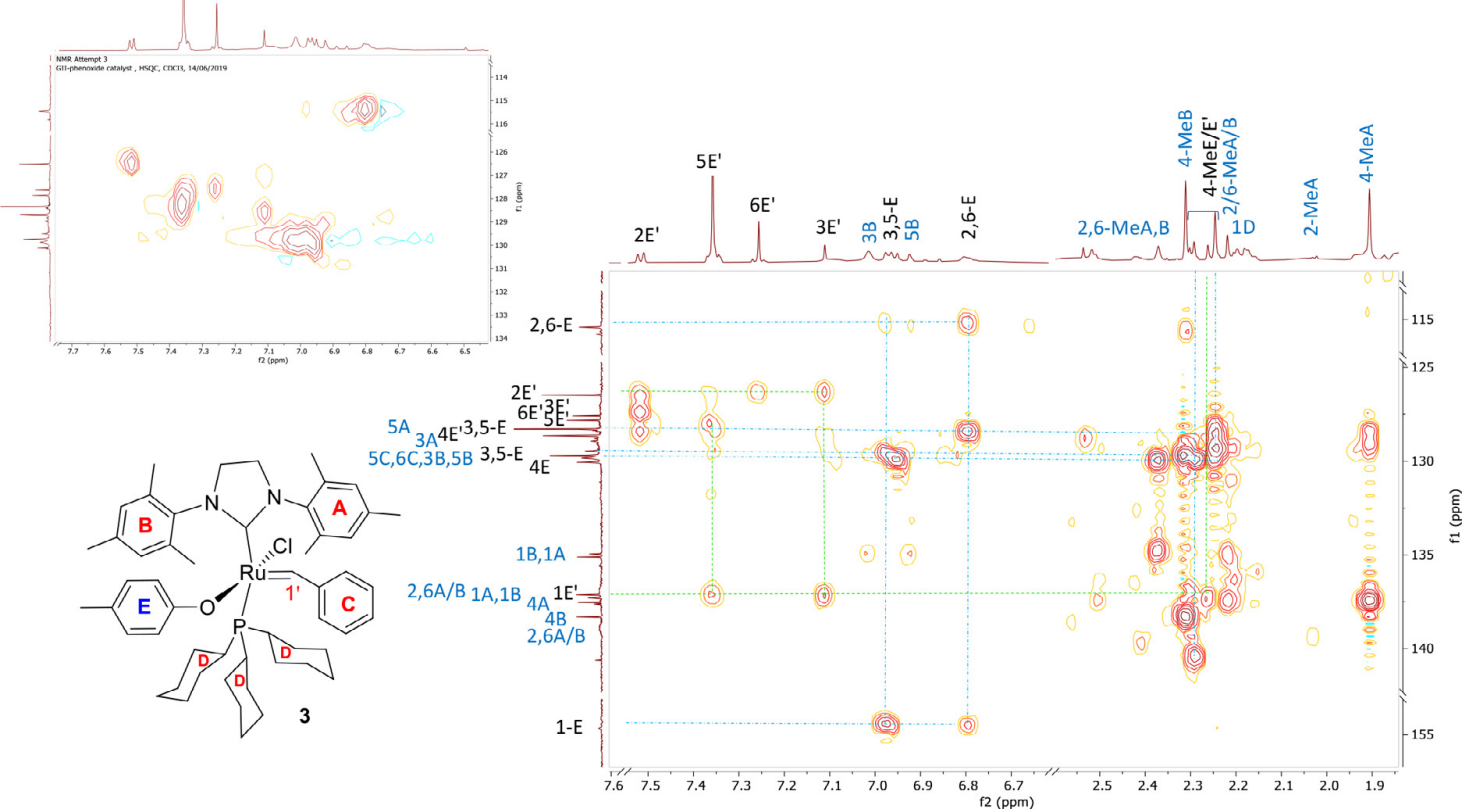
HSQC (insert) and HMBC spectra of complex (**3**) measured in CDCl_3_.

**Fig. 5:**
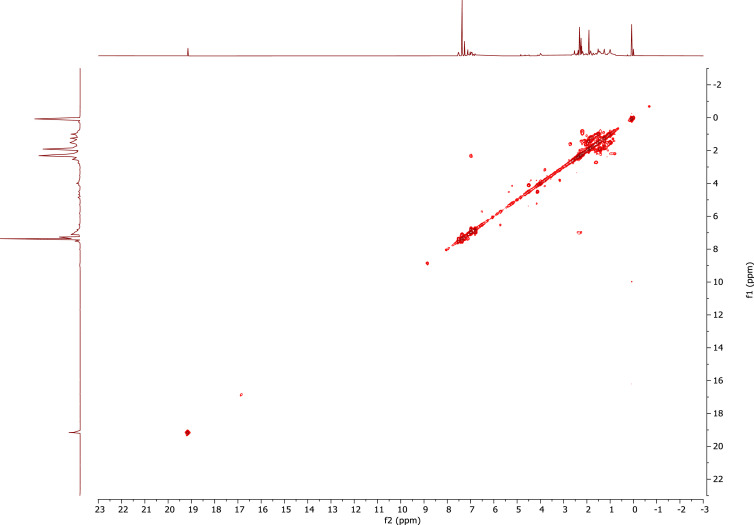
COSY spectra of complex (**3**) measured in CDCl_3_.

**Fig. 6:**
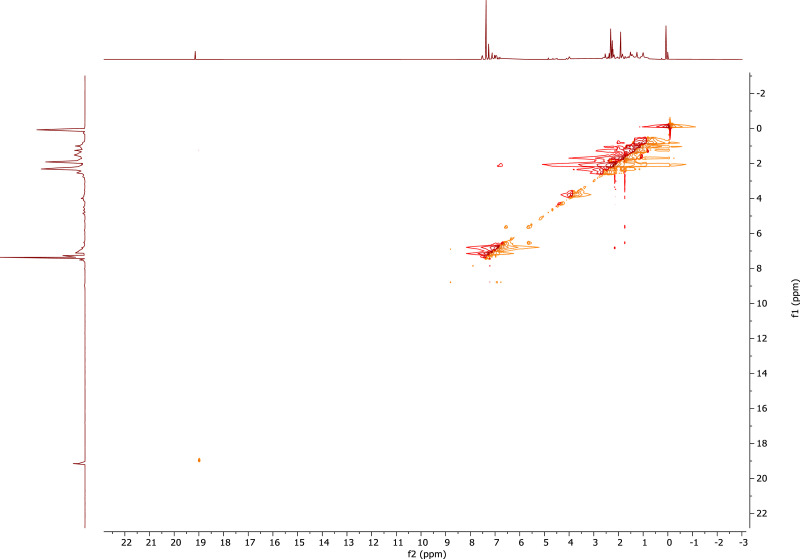
NOE spectra of complex (**3**) measured in CDCl_3_.

**Fig. 7:**
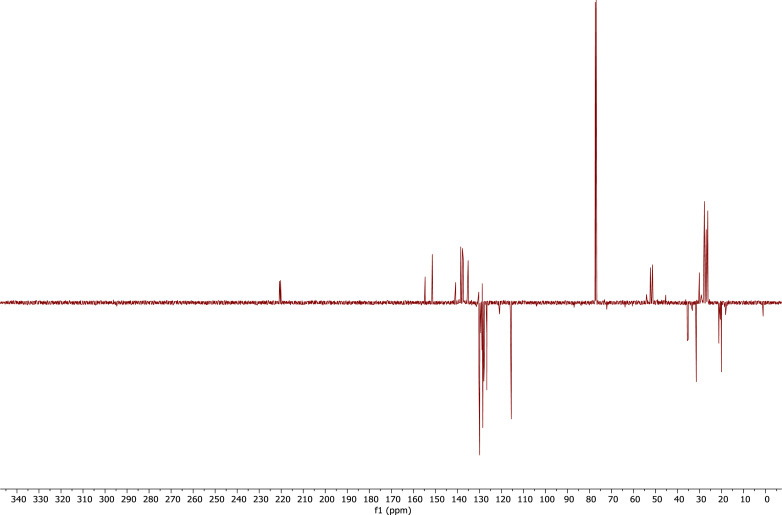
DEPT spectra of complex (**3**) measured in CDCl_3_.

A derivative between *p-*cresol (**1**) and Grubbs 2^nd^ generation catalyst (**2**) was prepared. The adduct, **3**, is a Grubbs 2^nd^ generation-*p*-cresolate derivative with a Ru-O coordination as a result of Cl - *p*-cresolate ligand exchange. The structure of **1-3** are presented in Fig. 1, with the structure of **3** showing the numerical labels used to assign the NMR data presented in Table 1. The ^1^H and ^13^C Nuclear Magnetic Resonance NMR resonances of **3** (see Figs. 2 and 3) were allocated in analogy to those of Grubbs second generation catalyst (**1**) [2,3] and by means of Heteronuclear single quantum coherence spectroscopy (HSQC) and Heteronuclear Multiple Bond Correlation (HMBC) experiments and are summarised in Table 1. The HSQC and HMBC spectra of complex (**3**) are presented in Fig. 4. Fig. 5, Fig. 6, Fig. 7 depicts the homonuclear correlation spectroscopy (COSY), Nuclear Overhauser effect (NOE) and Distortionless enhancement by polarization transfer (DEPTH) spectra of **3** measured in CDCl_3_. The raw data obtained from NMR instrument (^1^H, ^13^C, HSQC, HMBC, COSY, NOE and DEPT) was in the form of FID files which are difficult to understand without plotting. The raw data in the form of FID files were plotted using BRUKER TOPSPIN software which is presented in the form of images, shown in Fig. 2, Fig. 3, Fig. 4, Fig. 5, Fig. 6, Fig. 7. The raw NMR data are shared as supplemental files in the form of FID files and in Microsoft Excel Worksheet format.

**Table 1:** ^1^H and ^13^C NMR data of Ru(=CHC_6_H_5_)(OC_6_H_4_CH_3_)(Cl)(PCy_3_)_3_(H_2_IMes) (**3**) in CDCl_3_ at 25°C.

Position	^1^H δ (ppm)	m, J (Hz)	^13^C δ (ppm)	m, J (Hz)	HMBC correlations and other support
2	-		220.4	d, 77.6	
4	3.99	br s	52.2	d, 3.79	
5	3.80	br s	51.3	br s	
	3.99	br s	52.2	d, 3.79	
1′	19.14 16.84(trace) 16.80 (trace)	s	294.5 288.8 (trace)		1C, 2C, 6C
1 (A)			134.9 or 135.1		
2&6 (A)	-		139.5 – 138.5 and/or 137.0 – 136.5	m, m	
3 (A)	5.82	br s	128.9 & 128.4		4-CH_3_(A)
4 (A)	-		137.7 & 137.6		4-CH_3_(A)
5 (A)	6.72	br s	128.9 & 128.4		4-CH_3_(A)
2-Me (A)	2.15 – 1.96	m	19.0 – 18.2		Direct coupling
4-Me (A)	1.91	s	20.9		Direct coupling; C-3(A), C-5(A), C-4(A)
6-Me (A)	2.64 – 2.45	m	19.0 – 18.2		Direct coupling
1 (B)			135.1 or 134.9		
2&6 (B)	-		139.5 – 138.5 and/or 137.0 – 136.5	m, m	
3 (B)	7.01 (2H)	br s	129.9		2-CH_3_(B), 4-CH_3_(B)
4 (B)	-		138.4		4-CH_3_(B)
5 (B)	6.92	s	127.1		4-CH_3_(B)
2-Me (B)	2.76 & 2.37	br s	20.0		Direct coupling; C-1(B)
4-Me (B)	2.31	s	21.2		Direct coupling, C-4(B)
6-Me (B)	2.64 – 2.45	m	20.0		Direct coupling
1 (C)			151.2		
2 (C)	9.05 – 8.88	m	132.5 – 131.1	m	C-4(C)
3 (C)	7.15 – 7.05 a	m	126.5		
4 (C)	7.38 – 7.33^a^	m	128.4		C-3(C), C-5(C), C-6(C) and DEPT
5 (C)	7.15 – 7.05 a	m	130.1		
6 (C)	7.15 – 7.05 a	m	129.5		
1 (D)	2.22 – 2.14	m	29.4 – 28.7	m	
2, 3, 5, 6(D)	1.95 – 0.6	Various m	35.2	d, 40.1	
			31.4	d, 16.4	
			29.0	br d	
			27.7	d, 10.0	
			26.8	d, 11.6	
			26.3	d, 3.1	
4D			26.1	br s	
1 (E)	-		154.7		
2,6 (E)	6.84 – 6.69	m	115.4		C-1(E)
3,5 (E)	6.97 6.99 – 6.93	d, 8.0 m	129.8, 128.4		C-1(E)
4 (E)	-		129.5 - 130.1		DEPT
4-Me (E)	2.30 – 2.24	m	20.5 & 18.3 – 17.2	s and m	C-3(E), C-5(E), C-4(E)
1 (E')			137.1		
2 (E')	7.52	d, 7.4	126.5		
3 (E')	7.15 – 7.05 a	m	127.1		C-1(E'), C-2(E')
4 (E')			128.7		4-CH_3_(E') (and DEPT)
5 (E')	7.38 – 7.33^a^	m	127.9		C-1(E'), C-4(E')
6 (E')	7.28 – 7.23^b^	m	127.6		C-2(E')
4-Me (E')	2.30 – 2.24	m	20.5 & 18.3 – 17.2	s and m	

a Multiple peaks overlapping;

b Overlaps with CHCl_3._ A: mesityl ring *pi*-stacked with the benzylidene ring; B: second mesityl ring; C: benzylidene aromatic ring; D: cyclohexyl rings; E and E': *p*-cresolate moieties; 1ˈ: benzylidene; 2, 4 and 5: *N*-heterocyclic carbene ring

## 2 Experimental Design, Materials and Methods

Materials and methods to prepare the Grubbs 2^nd^ generation derivative **3**, which allowed the data to be presented here are describes in Ref [1]. In this article only the protocol used to record the NMR and UV-Vis data are provided.

### 2.1 Spectroscopic measurements

After removal of the solvent from the reaction mixture (in which **3** were prepared) under vacuo. The residue was dissolved in CDCl_3_ (0.6 mL) for NMR spectral analysis. ^1^H and ^13^C NMR measurements were recorded on a Bruker AVANCE II 600 FT NMR spectrometer at 278.15 K. The chemical shifts are reported relative to SiMe_4_ at 0.00 ppm for ^1^H and ^13^C. The ^1^H NMR spectra were recorded at 600.26 MHz and ^13^C NMR spectra at 150.95 MHz. HMBC and HSQC was used to assign the NMR signals. 8 Scan (TD1 = 512, TD2 = 2048) were recorded for both HMBC and HSQC with a relaxation time delay of 1.5 s. The acquisition time for the HSQC was 0.0655 s for the ^1^H and 0.00486 s for the ^13^C. The spectral width of the HSQC for the ^1^H is 26.0 ppm / 15 625 Hz and 348.63 ppm / 52 631 Hz for ^13^C. The acquisition time for the HMBC was 0.131 s for the ^1^H and 0.00486 s for the ^13^C. The spectral width of the HMBC for the ^1^H is 26.0 ppm / 15 625 Hz and 348.63 ppm / 52 631 Hz for ^13^C. For the COSY, NOE and DEPTH NMR a relaxation time delay of 1.5 s was used. The acquisition time for the COSY was 0.0655 and 0.033 s for the ^1^H. The spectral width of the COSY for the ^1^H is 26.0 ppm / 15 625 Hz and 25.9 ppm / 15607 Hz. The acquisition time for the NOE was 0.131 and 0.016 s for the ^1^H. The spectral width of the NOE for the ^1^H is 26.0 ppm / 15 625 Hz and 25.9 ppm / 15607 Hz. The acquisition time for the DEPT was 0.612 s for the ^13^C. The spectral width of the DEPT for the ^13^C is 354.85 ppm / 53571 Hz.

## Declaration of Competing Interest

The authors declare that they have no known competing financial interests or personal relationships that could have appeared to influence the work reported in this paper.

## Appendix Supplementary materials

Image, application 1

Image, application 2
